# Aβ42 pentamers/hexamers are the smallest detectable oligomers in solution

**DOI:** 10.1038/s41598-017-02370-3

**Published:** 2017-05-30

**Authors:** Martin Wolff, Bo Zhang-Haagen, Christina Decker, Bogdan Barz, Mario Schneider, Ralf Biehl, Aurel Radulescu, Birgit Strodel, Dieter Willbold, Luitgard Nagel-Steger

**Affiliations:** 1 Institut für Physikalische Biologie, Heinrich-Heine-Universität Düsseldorf, 40225 Düsseldorf, Germany; 2 Institute of Complex Systems, Structural Biochemistry (ICS-6), Forschungszentrum Jülich, 52425 Jülich, Germany; 3 Jülich Centre for Neutron Science & Institute of Complex Systems, Neutron Scattering (JCNS-1&ICS-1), Forschungszentrum Jülich, 52425 Jülich, Germany; 4 Jülich Centre for Neutron Science, Outstation at MLZ (JCNS-MLZ), Forschungszentrum Jülich, 85747 Garching, Germany; 5 Physikalische Biochemie, University Potsdam, 14476 Golm, Germany

## Abstract

Amyloid β (Aβ) oligomers may play a decisive role in Alzheimer’s disease related neurodegeneration, but their structural properties are poorly understood. In this report, sedimentation velocity centrifugation, small angle neutron scattering (SANS) and molecular modelling were used to identify the small oligomeric species formed by the 42 amino acid residue long isoform of Aβ (Aβ42) in solution, characterized by a sedimentation coefficient of 2.56 S, and a radius of gyration between 2 and 4 nm. The measured sedimentation coefficient is in close agreement with the sedimentation coefficient calculated for Aβ42 hexamers using MD simulations at µM concentration. To the best of our knowledge this is the first report detailing the Aβ42 oligomeric species by SANS measurements. Our results demonstrate that the smallest detectable species in solution are penta- to hexamers. No evidences for the presence of dimers, trimers or tetramers were found, although the existence of those Aβ42 oligomers at measurable quantities had been reported frequently.

## Introduction

An increasing number of human diseases are characterized by the accumulation of specific protein aggregates found in proteinaceous depositions with a common structural motif, which is characterized by a cross-β-sheet architecture called the amyloid fold^1^. While these amyloid fibril folds are already clarified for a number of proteins (reviewed in ref. 2), little is known about the first stages of the amyloid formation process, where dynamic, heterogeneous and often toxic intermediates are most likely populated. Such oligomeric species appear to be currently elusive to high-resolution structural methods such as solution based NMR or X-ray crystallography. Aside from the size limit of solution based NMR, the rather low fraction of the quested oligomer, its transient nature and heterogeneity renders NMR measurements at least extremely difficult^3^. Only with stabilized oligomeric preparations such studies have been feasible thus far^4^. Remarkably, Kotler *et al*. recently succeeded in the structural characterization of an Aβ40 oligomer of 5 to 15 nm size contributing to only 7% of the sample by magic angle spinning recoupling ^1^H-^1^H NMR experiments^5^. The above mentioned solution heterogeneity also interferes with the formation of diffracting crystals as required for X-ray crystallography.

In the case of Alzheimer’s disease (AD), which is the most common form of dementia worldwide, the accumulation of aggregated amyloid β protein (Aβ) in brain tissue is one of the disease hallmarks^6^. Aβ is generated as a proteolytic fragment from the transmembrane amyloid β-protein precursor (APP). Among the various isoforms that arise from variation of the location of the termini or by post-translational modification, the 42 amino acid long isoform (Aβ42) is the main constituent of extracellular senile plaques found post mortem in AD brains^7^.

Soluble Aβ oligomers, rather than the deposited fibrillar forms, are considered by many to be responsible for neurodegeneration in AD patients^8, 9^. The smallest Aβ assemblies reported in the literature are dimers, which exist in body fluids and brain tissues of AD patients^10, 11^. It should be noted that the finding of brain derived neurotoxic dimers by Shankar *et al*.^10^ had been refuted by later work^12^. Several studies of *in vitro* and *in vivo* experiments indicate the existence of a monomer-dimer equilibrium for Aβ^13, 14^. In photo-induced cross-linking of unmodified proteins (PICUP) experiments using Aβ, covalently linked dimers and other larger oligomeric species^12^ were detected for Aβ42 and for Aβ40, the shorter isoform missing the last two c-terminal residues^15, 16^. While for Aβ40 trimers and tetramers were the abundant species, for Aβ42 penta-/hexamers and also dodecamers and octadecamers could be detected^16^. Based on the PICUP technique the involvement of a penta- to hexameric oligomers in the Aβ42 assembly process had been reported in several studies^17–19^. Additionally, non-cross-linked Aβ oligomers have been identified in polyacrylamide gel electrophoresis as sodium dodecyl sulphate (SDS)-resistant bands and interpreted as dimers to tetramers^20^. Cultured neuronal cells were found to release small Aβ oligomers, among which the dimer represents a major species, whose size was determined by SDS-PAGE combined with western blotting^21–23^. Furthermore, dimers were found along with monomers and trimers as stable components, which could be purified by size exclusion chromatography under denaturing conditions, in neuritic plaques obtained from AD brain material^24^. Other studies have identified Aβ paranuclei as penta- or hexamers, which were detected by mass spectrometry based techniques^25, 26^. Recently, characterization of an enriched Aβ42 oligomer by NMR combined with AFM was achieved^27^. Further information on Aβ oligomers can be found in recently published reviews^28–34^.

Considering the plethora of different experimental approaches, even those techniques which produced the most consistent results on the early assembly states of Aβ42, i.e., PICUP and ISM-MS, possess some shortcomings which should be addressed for the sake of gain in knowledge. Crosslinked oligomers are only well reported by PICUP if all subunits become stably connected to withstand SDS-denaturation prior to SDS-PAGE. Detergence below or above their critical micellar concentration might stabilize assemblies by shielding hydrophobic regions, which would not be stable in hydrous solutions. Ionization coupled with transfer to the gas phase as required for mass spectrometry, on the other hand, might be a selective as well as destructive process. Recently, a critical evaluation of PICUP followed by SDS-PAGE analysis performed by IMS-MS challenged the penta- to hexameric building block in Aβ42 aggregation^35^. A clear understanding of the early steps in the self-association of Aβ is critical in order to understand the formation of toxic oligomeric species and the initiation of fibrillisation in Alzheimer’s disease. We therefore chose an approach with high detection sensitivity, which is applicable to proteins in solution, for the characterization of the smallest oligomers of Aβ.

Recently, sedimentation velocity (SV) analysis, which is an application of analytical ultracentrifugation (AUC), was shown to be a highly sensitive detection method for trace amounts of aggregates in protein samples. Sedimentation velocity centrifugation allows distinguishing multiple sedimenting species in free solution while maintaining reversibly formed complexes in a bath of their components at all times. This permits the study of self-association as well as heterogeneous protein interactions^36^. Using SV, <1% aggregates in a sample can be reliably detected, provided instrument and equipment are handled with sufficient care^37^. SV analysis can determine the size- and shape distributions for self-assembling proteins^38–41^. Due to the fractionating property of the sedimentation process, with large particles sedimenting before small particles, the method is resistant to the presence of large particles such as unresolved protein aggregates or dust particles. The method is characterized by an excellent signal-to-noise ratio. The evolution of the shape of the formed sedimentation boundary over time reveals not only information about the sedimentation velocity but also about the diffusion properties of the analytes^42, 43^. Importantly, the method requires no interactions of Aβ with surfaces that might induce aggregation. SANS is a well-established, non-destructive method to examine structure on length scales of 1 to 1000 nm. While only one report on a SANS study of full length Aβ was found in PubMed^44^, which is about the characterization of Aβ40 at acidic solvent conditions, some more reports exist on small angle X-ray scattering (SAXS) studies of Aβ42^45, 46^. X-ray scattering has the disadvantage of being destructive to proteins and the generation of radicals might impair the aggregation process. In a previous study, we demonstrated the applicability of SANS using monomeric Aβ^47^. While in SV the hydrodynamic properties of macromolecules are evaluated, yielding the hydrodynamic radius, in SANS the properties of macromolecules as neutron scatterers are evaluated yielding also the radius of gyration, aside from information about size, shape and interactions. Because the two solution-based methods are exploiting different physical properties they are ideally suited to complement each other.

By SV analysis, we previously demonstrated the existence of discrete oligomeric species in the *s*-value range from 4 to 15 S during the lag phase of fibril formation^48^. Analysis reproducibly yielded two Aβ42 assembly species at low salt conditions and physiological pH, independent of the total Aβ42 concentration; one corresponding to a 12-mer and the other to an 18-mer of Aβ42. These sizes suggest the involvement of a trimeric or hexameric building block. A pentameric to hexameric building block could be evaluated by PICUP for Aβ42 and has been defined by Bitan *et al*. as paranucleus^15, 16, 44^. We interpreted the recently observed gap in the determined *s*-value distributions between 0.69 S, corresponding to the Aβ42 monomer and 4 S as the range for the nucleus size^48^. In this *s*-value region corresponding to molecular weights between 5 and 55 kDa, we hypothesize further on-pathway to fibril reaction intermediates. Here, we aim to close this gap by combining two techniques for the structural characterization of macromolecules in solution, namely SV analysis and small angle neutron scattering (SANS). Because the two solution-based methods are exploiting different physical properties they are ideally suited to corroborate each other in order to provide important information about the first steps of the nucleation process. In addition, we further complement our experimental results by including results from molecular dynamics (MD) simulations of Aβ42 assemblies, which bridge our SV and SANS results. This combined approach reveals clear experimental evidence for a penta- or hexameric assembly of Aβ42 in solution.

## Results

The goal of this study is to identify metastable intermediates during the aggregation of Aβ42 in solution and thus to elucidate the initial steps of Aβ42 assembly.

### Sedimentation velocity centrifugation allows sensitive detection of Aβ oligomers in solution

The first step in searching for small Aβ assemblies was to identify the smallest species, i.e., the dimers, and prove that SV analysis can discriminate them from monomers. As a model, a stable synthetic Aβ40 dimer was generated from a monomer with a cysteine residue at position 0, which formed an intermolecular disulphide bridge under oxidizing conditions. SV analysis revealed that the Aβ monomer and the covalently linked dimer are sufficiently different, with *s*-values of 0.65 and 0.9 S, respectively (Fig. S2). None of the SV experiments performed for non-linked Aβ42 provided evidence of an Aβ42 dimer in solution. If Aβ42 dimers or trimers were in rapid equilibrium with the monomers, one would expect that at higher Aβ42 concentrations the equilibrium were shifted from monomers towards the dimers and trimers. For such a sample, the peak in *c*(*s*) would not represent a single *s*-value species, but a so-called reaction boundary. A diagnostic for the identification of such a reaction boundary would be a shift towards higher *s*-values at higher Aβ42 concentrations or at least peak broadening. The superposition of the *c*(*s*) peaks for the monomer from different data sets revealed no Aβ42 concentration dependent shift in *s*-value nor peak broadening of the *c*(*s*) signal at 0.62 S. We thus conclude that the dimer fraction is below the detection limit.

### The smallest detectable assembly state in the SV experiments is a penta- to hexamer

Experimental evidence for oligomeric species between trimers and 18-mers were found mainly from SDS-PAGE analysis after PICUP^15, 16^ and mass spectrometry studies. For example, a trimer is postulated as the building block for the Aβ*56 aggregate found in *in-vivo* studies by Lesné and colleagues^49, 50^, and trimers along with tetramers were also detected by Bernstein *et al*.^17, 51^. We decreased the total Aβ42 concentrations to the low micro- to nanomolar concentration range in our SV experiments, expecting that at Aβ42 concentrations below the critical concentration for the formation of higher-order structures, the assembly of larger assemblies from small oligomers is suppressed. The necessary increase in detection sensitivity was gained by using AUC coupled with a fluorescence detection system. Thus, a lower limit of about 100 nM fluorophore labelled Aβ42 was achieved. For the position of the fluorescence label we chose the N-terminus of Aβ, because the N-terminus is not primarily involved in intermolecular contacts required for aggregate formation^52^ and similar conjugates had been successfully used before^53^. Additionally, those regions of Aβ42, which had been identified as critical for paranucleus formation, i.e. the central hydrophobic core as well as the C-terminal residues^15^, are distantly located from the fluorophore. A linker further separates the fluorophore from the Aβ42 peptide and introduces flexibility. Moreover, charged fluorophores also possess a smaller propensity to stack on each other than uncharged fluorophores. From our AFM measurements (Fig. S3) we concluded that the fluorescence label neither prohibits fibril formation nor leads to extensive amorphous aggregate formation. Nevertheless, it cannot be fully excluded that the presence of a fluorescence label at the N-terminus favours certain paranuclei conformations, which would be less or not at all populated by the wild-type Aβ42 peptide. Thus, we conclude that the application of the fluorophore-labelled conjugate allow us to expand our measurements into the submicromolar Aβ42 concentration range and to draw conclusions that can be transferred with the above mentioned restrictions to wild-type Aβ42.

**Figure 1:**
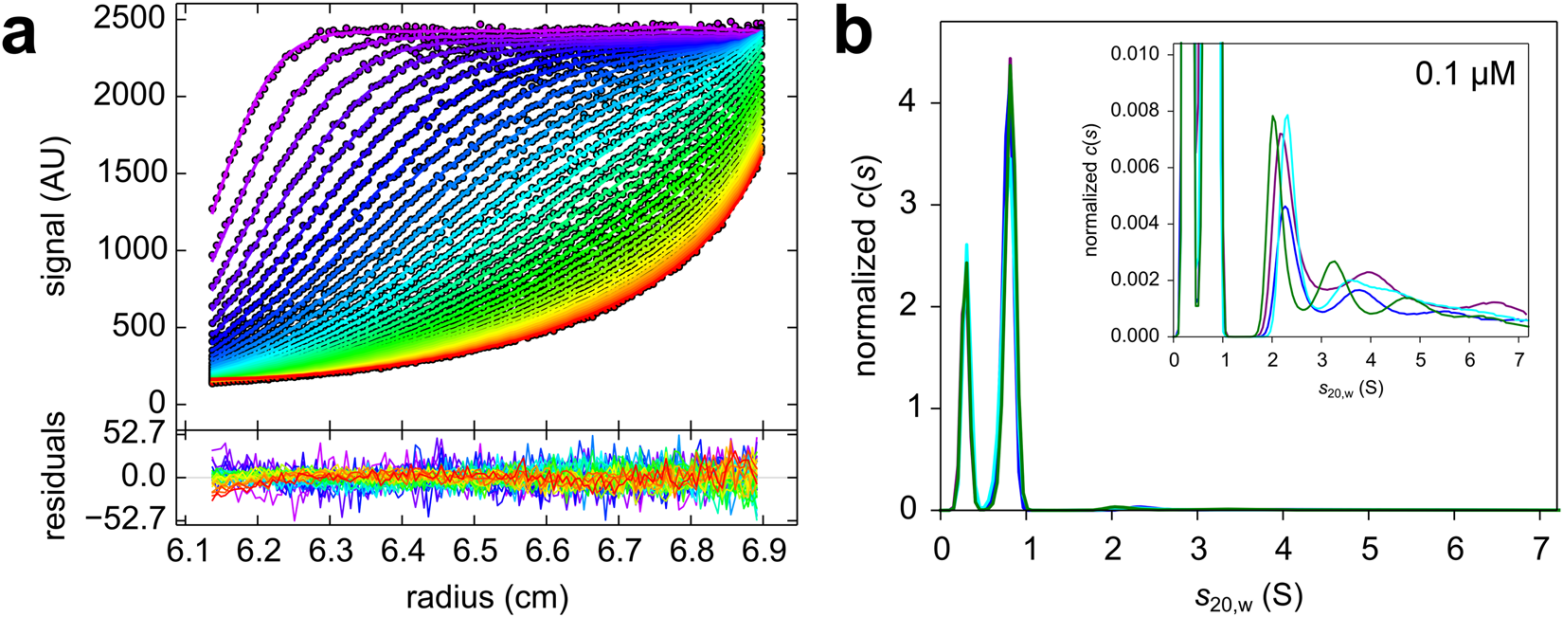
SV analysis of 0.1 µM AF488-Aβ42 with fluorescence detection reveals the existence of small oligomers in addition to the monomer. (**a**) Raw data of SV experiment with overlaid lines for the fitted data, showing a signal intensity of about 2500 RFU. Only every third scan, as well as every third data point is shown for clarity. In the box below the corresponding residuals within the fitted data range are shown. Rmsd for this sample was 11.86. (**b**) Area normalized *c*(*s*) results for four independent samples of 0.1 µM AF488-Aβ42 have been superimposed in the graph. The 0.8 S species is assigned to the monomer, the peak at 0.3 S is the result of either incomplete removal of unincorporated dye or the dark count rate or a combination of both. The inset shows the magnification of *c*(*s*) distributions. Sedimentation was performed at 60,000 rpm, 20 °C.

SV analysis is a technique, which recently gained special relevance in the determination of aggregate concentration in samples of pharmaceutically relevant proteins^54–57^. According to theoretical considerations at high signal-to-noise ratios of absorbance and fluorescence detection, a superior sensitivity for traces well below 1% of total signal is achievable in sedimentation velocity experiments^57^. For example; this would correspond to 0.01 absorbance units of an oligomer in the presence of 0.99 absorbance units of the monomer at a suitable wavelength. The limit of detection (0.2% of total signal) and the limit of quantification (about 2.8% of total signal) have been determined from practical studies on a dimeric antibody^37^. For confirmation of the detected high molecular weight species, the calculation of the *c*(*s*) distribution was extended by applying a Bayesian approach followed by F statistics, as reported recently by Wafer *et al*.^54^. After repeating the *c*(*s*) calculation by alternating between the simplex and Marquardt-Levenberg algorithms until no further change in rmsd was observed, the *c*(*s*) model is exchanged by a model of non-interacting species. This model can then be tested by F-statistics for the significance of the high molecular weight species. This was achieved by calculating a critical relative root mean square deviation (rmsd) value based on the model including the oligomeric species. A species is considered as a significant part of a sample if the rmsd of a fit excluding this species exceeds the critical relative rmsd. Thus the significance of the oligomeric species was shown for four data sets by observing an increase of the rmsd above the critical rmsd upon removal of the larger species (Fig. 1a,b).

The total Aβ42 concentration was varied from 0.1 μM to 160 μM. All SV measurements were performed with Aβ42 incubation times of ~2 h before SV centrifugation. The *s*-value for the monomeric AF488-Aβ42 was determined as 0.80 ± 0.01 S, which is slightly higher than the *s*-value 0.6 ± 0.02 S found for the unlabelled monomer (Table 1). The monomer accounts for 61% of the total fluorescence signal. The *s*-values of the smallest detectable oligomers in this experiment were determined as 2.32 ± 0.14 S for AF488-Aβ42 and 2.56 ± 0.32 S for Aβ42. The smaller *s*-value for AF488-Aβ42 oligomer in comparison to the unlabelled Aβ42 oligomer could be explained by an increase of the frictional coefficient *f* or shape factor *f*/*f*_0_ for AF488-Aβ42 due to the fluorophores protruding away from the oligomer. For the monomer, which is unstructured in aqueous solution^46^, the increase in mass seems to be the dominating effect leading to an increase of the *s*-value for AF488-Aβ42. In Fig. 1b the *c*(*s*) distributions for four independent samples of 0.1 μM AF488-Aβ42 dissolved in 10 mM phosphate buffer are shown. At this low concentration aside from the monomer and the residual free dye only a small fraction of small oligomeric species were detected. These oligomers are dominated by a species between 2 S and 3 S, which represents the smallest *s*-value of the oligomer distribution. The fraction of this species accounts for about 1.4% of the total signal. After subtracting the contribution of the free dye the value increases to 2% of total AF488-Aβ42. In order to evaluate whether by changing the temperature the equilibrium could be shifted from monomers to penta-/hexamers, we performed SV experiments at different temperatures between 10 and 30 °C. However, variation of the experimental temperature in this range did not lead to measurable changes in the oligomeric fractions. To exclude the possibility of contamination or that we observe a covalently bonded aggregate species, we treated the sample with 6 M guanidine hydrochloride. Under these denaturing conditions this oligomer species disappeared (data not shown).

**Table 1:** Experimentally determined sedimentation coefficients for Aβ42 monomer and small oligomer.

	Monomer (S)*	Small Oligomer (S)*
Aβ42	0.62 ± 0.02	2.56 ± 0.32
AF488-Aβ42	0.80 ± 0.01	2.32 ± 0.14

^*^Sedimentation coefficients for the monomeric and small oligomeric species of Aβ42 were determined from *c*(*s*) distributions. Weight averaged *s*_20,w_-values and standard deviations were calculated from at least 4 independent sample preparations.

**Figure 2:**
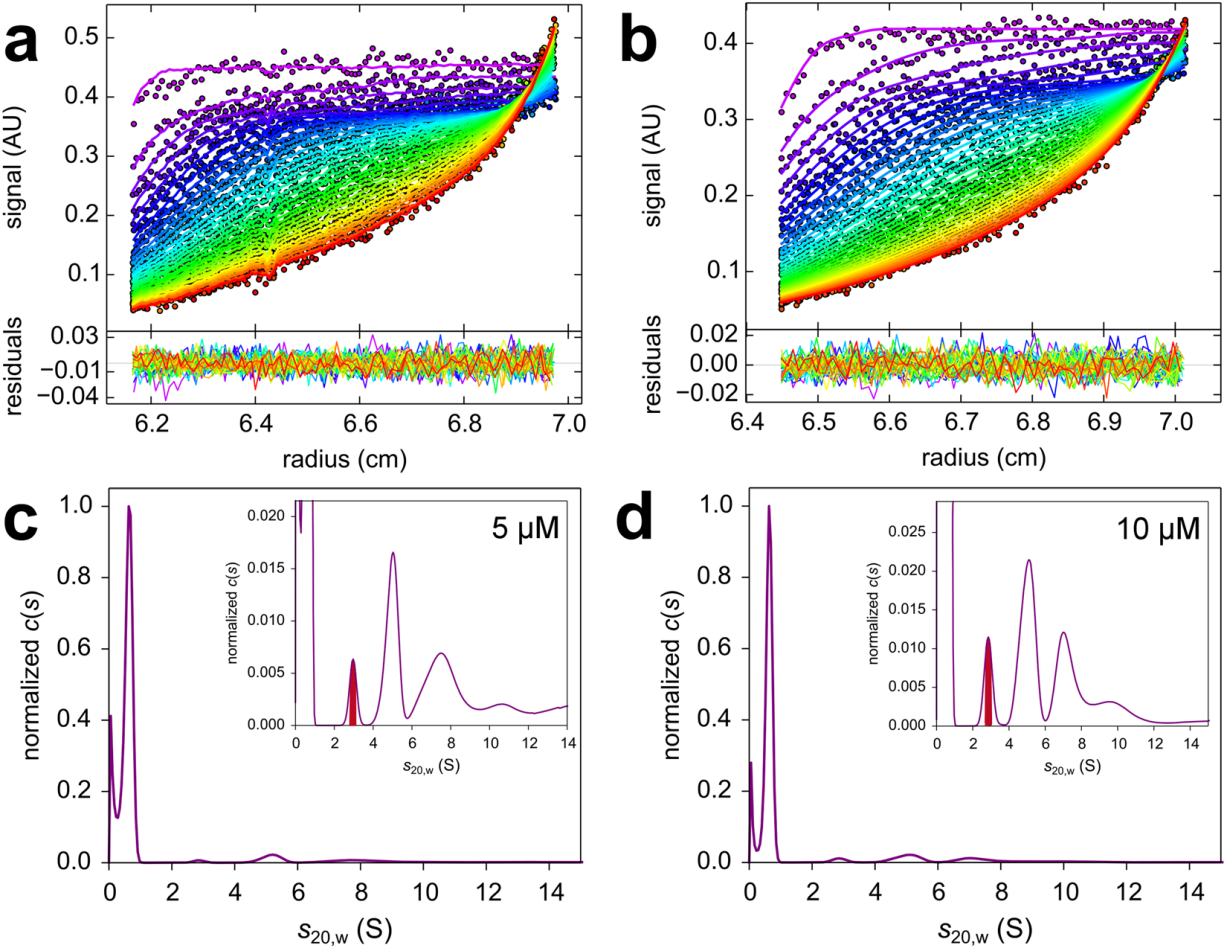
The SV analysis at low micromolar Aβ42 concentrations yields the existence of the small oligomer between 2 S and 3 S and a fraction of larger assemblies with *s*-values between 3 and 12 S. The raw data at 5 µM (**a**) and 10 µM Aβ42 (**b**) together with overlaid fit results and residuals are shown. The corresponding *c*(*s*) results for 5 µM (**c**) and 10 µM Aβ42 (**d**) are shown below. Sedimentation was performed at 50,000 rpm, 20 °C and monitored with absorbance detection. Detection wavelength for 5 µM was 208 and 220 nm for 10 µM Aβ42. The *c*(*s*) distribution was normalized to a maximum *c*(*s*) value. The insets show the distributions with an expanded *y* scale.

**Figure 3:**
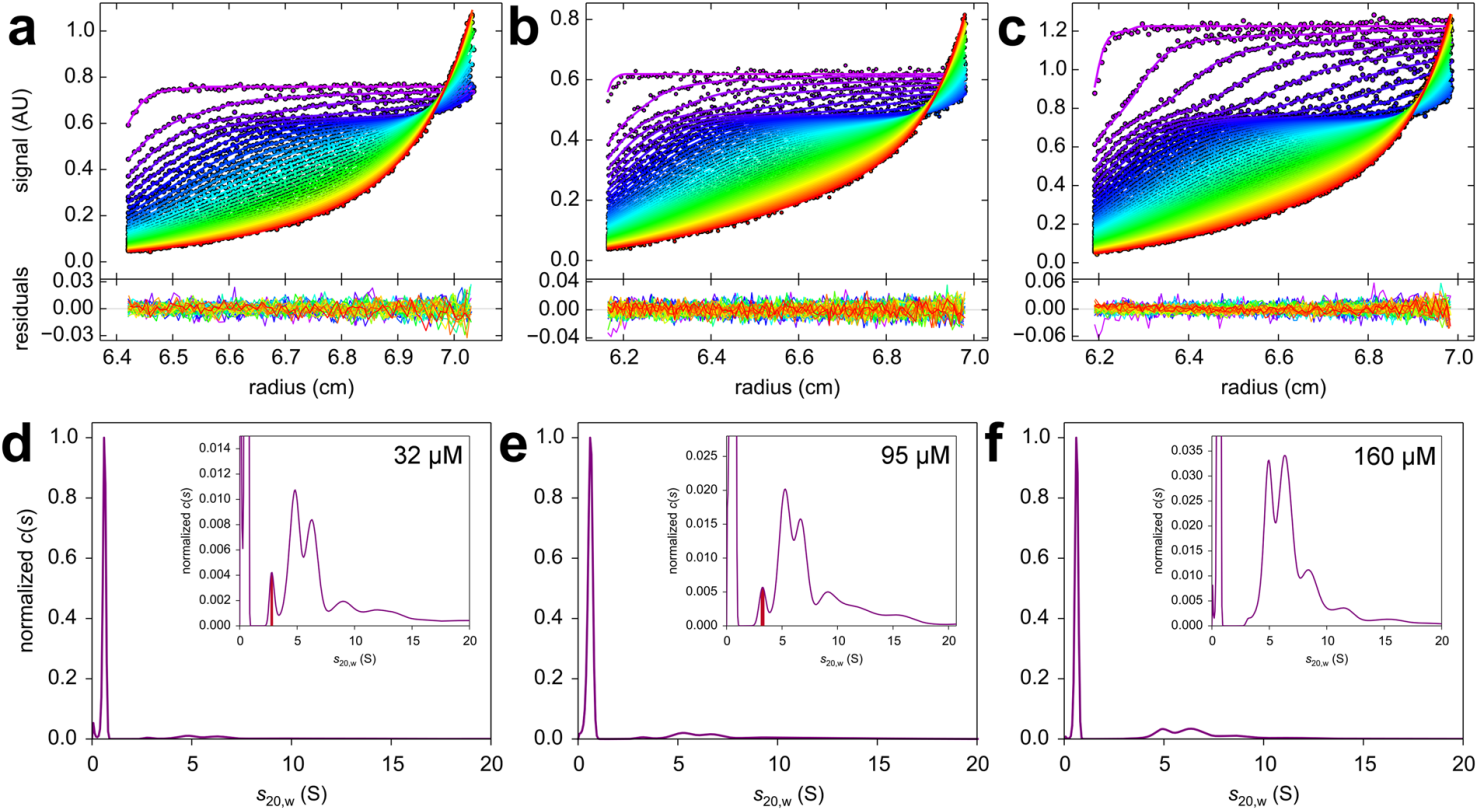
SV analysis at high Aβ42 concentrations. Measured data overlaid with fit data and corresponding residuals are shown for 32 μM (**a**), 95 μM (**b**) and 160 μM (**c**) Aβ42. Below, *c*(s) analysis reveals the existence of a high fraction of larger oligomeric species aside from the monomer and a comparably low fraction of the 2 to 3 S species for 32 µM (**d**), 95 µM (**e**) and 160 µM (**f**) Aβ42. Sedimentation was performed at 60,000 rpm, 10 °C and monitored with absorbance detection at 280 nm. Normalization was performed according to peak height of the monomeric Aβ42. The insets show the distributions with an expanded *y* scale.

Figure 2 shows the *c*(*s*) distributions obtained for 5 (Fig. 2a,c) and 10 μM Aβ42 (Fig. 2b,c). At these medium Aβ42 concentrations, larger aggregates become detectable in addition to the small oligomers. Figure 3 shows three *c*(*s*) distributions obtained for high Aβ42 concentrations, which demonstrate that the small oligomer is still detectable under these conditions, where larger aggregates have already formed. In Table 1, the mean *s*_20,w_-values for the monomer and for the small oligomers are summarized for Aβ42 and AF488-Aβ42.

In summary, SV measurements covering a concentration range between 0.1 μM and 160 μM consistently reveal the presence of a small oligomer at 2.56 S or 2.32 S for Aβ42 and AF488-Aβ42, respectively. According to MD simulations described below, these values correspond to Aβ42 pentamers or hexamers.

### Small angle neutron scattering studies of Aβ42 in solution corroborate the presence of penta- to hexamer species

**Figure 4:**
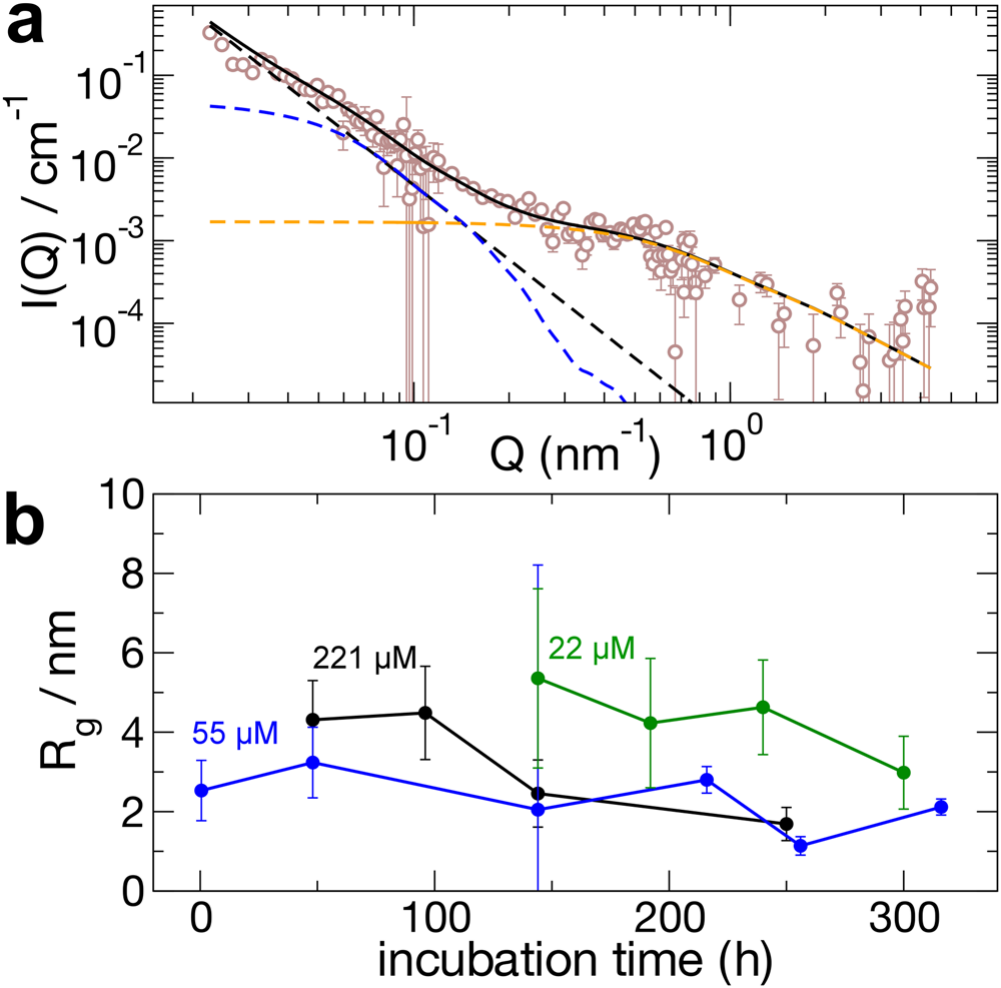
Results from SANS scattering experiments after incubation of Aβ42 at pH 7.4. Measurement and incubation was in 50 mM Napi plus 150 mM NaCl in 100% D_2_O at 7 °C. (**a**) Exemplary SANS scattering intensity after 0.5 h. Contributions to the scattered intensity arise from aggregates larger than 50 nm resulting in a power law (black broken line), mid-size aggregates modelled as ellipsoid of revolution (blue broken line) and small oligomers (orange broken line), which are modelled by a Beaucage function. A vanishing background of monomers is modelled as a Beaucage function with fixed *R*_*g*_ = 1 nm. The combined fit result is shown as a black solid line. Ellipsoids have radii between 50 and 10 nm. (**b**) The radius of gyration of small oligomers from SANS analysis after incubation at concentrations of 221 μM (black), 55 µM (blue) and 22 µM (green).

In contrast to SV with fluorescence detection, SANS measurements do not require dye labelling; although, an equivalent D_2_O buffer is used to reduce buffer scattering. For the relative low concentrations used in the aggregation studies, a long counting time was required to obtain reasonable statistical accuracy because the detected signal at larger wave vectors is two orders of magnitude below the buffer scattering. To slow down the aggregation process and reduce measurement times to ~8 h for several detector distances without significant change in the sample, the measurements have been performed at 7 °C. SANS measurements have been performed for Aβ42 with incubation times between 0.5 and 316 h (Fig. 4) for concentrations of 221 μM (1 mg/ml), 55 μM 0.25 mg/ml) and 22 μM (0.1 mg/ml).

SANS measurements show that even at low temperature and relative low concentrations aggregates of all sizes are formed after the addition of buffer to the dry Aβ. This in in contrast to SV experiments, where at the speed applied to study the small oligomers larger oligomers, protofibrils or fibrils are not detectable due to their fast sedimentation. The scattering intensity of the samples did not change measurably within 10 h as monitored by dynamic light scattering measurements (data not shown). The measured intensities after buffer subtraction were analysed by a model adding contributions of larger aggregates and complexes, midsize aggregates, small oligomers and monomers as shown in Fig. 4a. Except for the monomer, the populations of the different assembly states were determined in accompanying measurements by dynamic light scattering (Fig. S4). Large aggregates or complexes, such as assemblies of fibrils with radii of gyration (*R*_*g*_) larger than 50 nm contribute at low *Q* by a power law $\sim {Q}^{-d}$ with *d* between 5/3 and 3 as expected for networks/gels^58^. Midsize aggregates, such as protofibrils or fibrils were modelled by an ellipsoid of revolution with *R*_*g*_ between 50 and 10 nm^1^. Small oligomers were modelled by a Beaucage model $(F({R}_{g},\,d))$ providing the *R*_*g*_ of the small aggregates with fixed dimensionality^59^. Monomers contribute with a fixed *R*_*g*_ = 1 nm as a background contribution and cannot be resolved. Small oligomers with *R*_*g*_ between 1 and 5 nm were observed at different concentrations throughout the experiments (Fig. 4b).

It should be emphasized that we observe oligomers and aggregates of different sizes, which are well separated in the SANS signal. If objects of all sizes were present in the sample, we would observe an undefined, decaying scattering intensity without a shoulder. Importantly, the small oligomers (identified as hexa-/pentamers, see below) can only be observed if species between the small oligomers and monomers are significantly missing and do not contribute intensity above the shoulder at ~0.6 nm^−1^. If smaller oligomers than pentamers were present, the scattered intensity could not be described by a Beaucauge function separated from the monomer background. Nevertheless, the presence of smaller species cannot be completely ruled out, but is limited to amounts which contribute to the scattering signal at the scale of the noise of the measurements.

### MD simulations provide size and shape information about early oligomeric species

**Figure 5:**
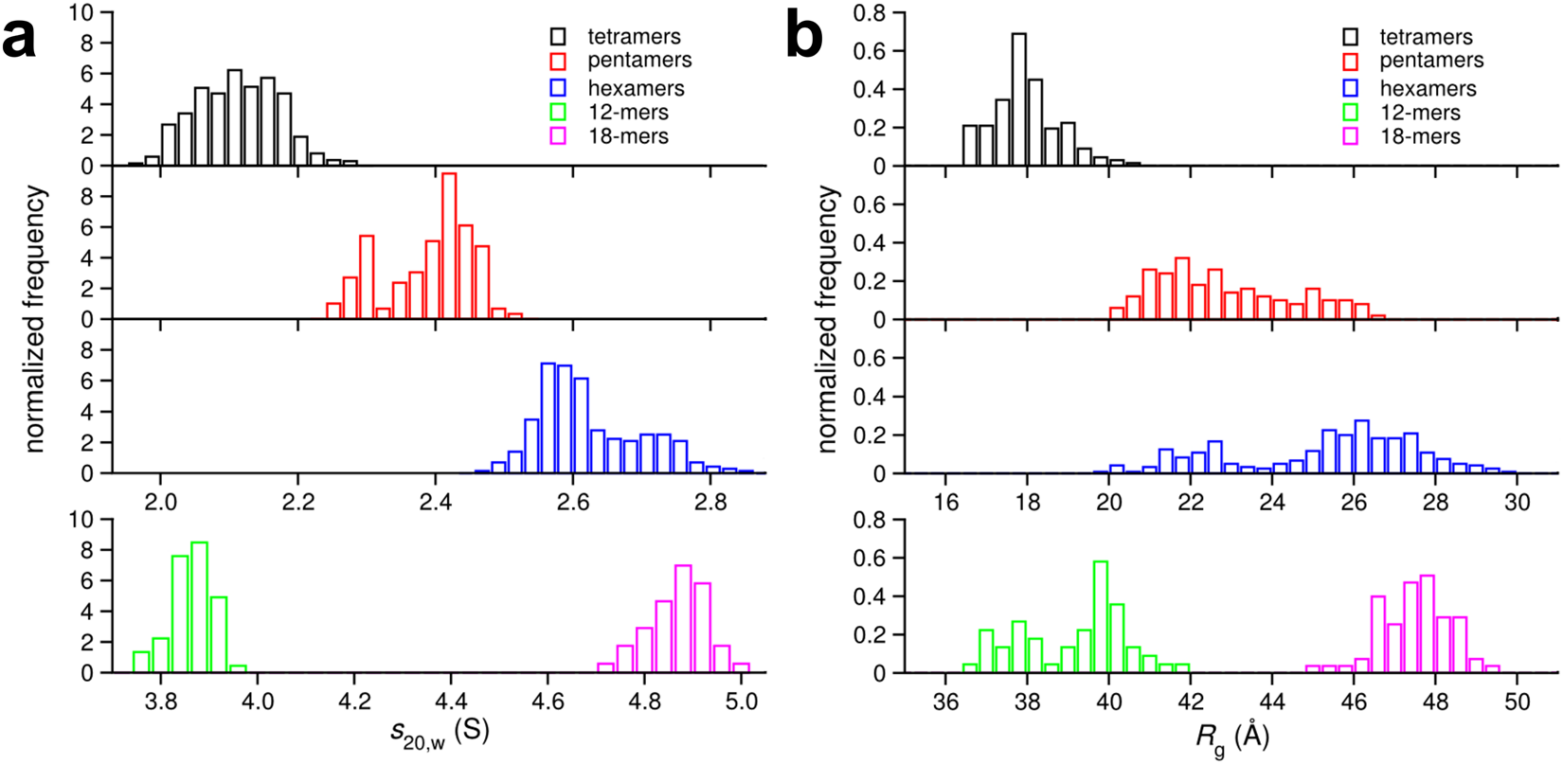
Calculated sedimentation coefficients and radii of gyration of Aβ42 oligomers obtained from MD simulations. (**a**) Normalized distributions of sedimentation coefficients for Aβ42 tetramers (black), pentamers (red), hexamers (blue), 12-mers (green), and 18-mers (magenta). (**b**) Normalized radii of gyration for the ensembles of Aβ42 tetramers (black), pentamers (red), hexamers (blue), 12-mers (green), and 18-mers (magenta). All values for tetra- to hexameric Aβ42 oligomers were calculated with the program HydroPro._ENREF_69 Corresponding weight averaged *s*-values are summarized in Table 2.

From the *R*_*g*_ values determined from the SANS data it is not possible to determine the size of the oligomers. Therefore, in order to compare the oligomer sizes observed in the SV experiments with the *R*_*g*_values obtained from the SANS experiments, we performed five independent all-atom MD simulations, yielding a total simulation time was 1 μs, of twenty Aβ42 monomers inserted in a cubic box at a solute concentration of ~80 µM^60^. While this concentration is considerably larger than that used in the SV experiments, it should be noted that, to the best of our knowledge, it is lower compared to all other atomistic simulations studying Aβ aggregation. Moreover, as the current simulation results are used to provide *R*_*g*_ and *s*-values for the different oligomer sizes, their relative population as sampled in the simulations is not important. Instead, of importance is that the oligomer structures are sampled realistically. In order to check that the oligomer structures are not affected by the high peptide concentration or the use of an implicit solvent, we performed additional MD simulations of isolated dimers, trimers and tetramers using an explicit water model. The resulting oligomer structures have similar *s*- and *R*_*g*_ values and also exhibit similar structures as those obtained from the simulations involving twenty Aβ peptides. Thus, it can be concluded that our MD results are not affected by the rather high peptide concentration used in the current simulations. First, we estimated the sedimentation coefficients of the modelled Aβ42 oligomers. Normalized distributions of the sedimentation coefficient for different oligomer orders are shown in Fig. 5a. The resulting weight averaged *s*-values for the calculated distribution are listed in Table 2. Tetramers displayed the lowest sedimentation coefficients with an average value of 2.09 ± 0.06 S, followed by pentamers with 2.38 ± 0.06 S and hexamers with an average value of 2.61 ± 0.07 S. For the current analysis we used 576 tetramers, 123 pentamers and 299 hexamers. The *s*-values obtained for the small oligomeric species collected in five MD simulations show a distribution, e.g., hexamers are found with *s*-values between 2.42 S and 2.90 S, which seems to approach a Gaussian like shape with higher sampling. The distributions reflect the heterogeneity of the simulated oligomers with regard to their surface area. Nevertheless the overlap between two adjacent oligomer sizes does not exceed 0.3 S units, which allows us to conclude that we identified pentamers and hexamers as smallest oligomeric species in our SV experiments.

**Table 2:** Average sedimentation coefficients for Aβ42 tetramers, pentamers and hexamers based on confirmations obtained from MD simulations.

Oligomer	Tetramer	Pentamer	Hexamer
*s*-value (S)	2.09 ± 0.06	2.38 ± 0.06	2.61 ± 0.07
No. of conformations	576	123	299

The number of conformations is the total number before the clustering algorithm was applied. All values were calculated with the program HydroPro._ENREF_75.

To correlate the *R*_*g*_ values determined from the SANS experiments, we calculated the ${R}_{g}$ for the ensemble of structures generated by our MD simulations (Fig. 5b). For the lowest Aβ concentration of 55 µM used in the SANS experiments, the constant presence of oligomers with *R*_*g*_ ~ 2.3 nm corresponds to pentamer and/or hexamer species. According to Fig. 5b, it is difficult to discriminate between pentamers and hexamers based on the *R*_*g*_ value only. At 221 μM the small oligomers have *R*_*g*_ values of around 4 nm at the beginning and after 120 h incubation we find smaller aggregates again with *R*_*g*_ ~ 2 nm, which probably correspond to a pentamer/hexamer (Fig. 4b). Interestingly, the largest radius of gyration calculated from SANS measurements of ~5 nm corresponds to assemblies of 18 Aβ42 peptides, according to MD simulations. At the earliest measurement time it was not possible to discriminate between different size classes because of the strong smearing between the various species. Importantly, the SANS studies revealed no species in size between the monomer and the putative pentamer/hexamer.

## Discussion

Our study shows evidence for the occurrence of small oligomeric assemblies built of five to six Aβ42 monomers in solution. This conclusion emerged from the application of two independent experimental approaches, which are based on different physical principles, SV analysis and SANS. The two approaches were bridged by MD simulations. The experimentally determined *s*-values of the smallest oligomers observed in SV experiments were compared to *s*-values calculated for a set of different oligomers generated by MD simulations. According to this comparison, a combination of pentamers and hexamers explains the best the experimentally observed *s*-values. From SANS measurements we calculated radii of gyration between 2 and 5 nm, which are in agreement with radii of gyration of assemblies ranging between pentamers and 18-mers calculated for the same set of MD simulated oligomers. Theoretically a sedimentation coefficient is informative on mass as well as shape of a molecular species, but since the detected oligomeric species represents only a very small fraction of the mixture the weight-average of *f*/*f*_0_ does not provide reliable information on mass or shape of the oligomer. Therefore, based on the experimentally determined *s*-values we calculated hydrodynamic radii assuming a tetra-, penta-, and hexamer (Supporting Eq. 1 and Supporting Table 2). Assuming that hydrodynamic radius and radius of gyration are sufficiently similar for the oligomer we concluded that the measured *s*-value can be best explained by a pentamer or hexamer.

These comparisons led us to a model of a penta- to hexamer for the small oligomeric Aβ42 species that would explain the best the SV and SANS results. Data interpretation was further complemented by MD simulations of Aβ42 assembly, which were performed as close as possible to the experimental conditions. Deviating buffer conditions in SANS experiments were shown to give comparable results in SV experiments (Fig. S5) with regard to oligomer formation.

Remarkably, in both the SV and SANS experiments only a small fraction of about 1 to 10% of total Aβ42 could be detected in the pentamer/hexamer state. The dominant Aβ42 species is the monomeric state and larger oligomers present only at higher concentrations. The consistently low fraction of the small oligomeric species over a broad range of Aβ42 concentrations, which is reported by both methods, is a strong indicator of its role as an important reaction intermediate with low population.

Within the accessible Aβ42 concentration range used it was impossible to shift the equilibrium towards higher fractions of the pentamer/hexamer assembly. Higher fractions are most probably prevented by a loss of the pentamer/hexamer to larger oligomers, once a threshold Aβ concentration has been reached. At concentrations below 10 µM, the pentamer/hexamer is the sole oligomeric species. At higher concentrations, the pentamer/hexamer appears to be in a steady-state equilibrium with the monomer and larger oligomeric species, representing a lowly populated reaction intermediate. This classifies the pentamer/hexamer to be an on-pathway intermediate of fibril formation.

Ahmed *et al*. reported the characterization of a penta- or hexameric Aβ42 with structural information from solution and solid-state NMR^27^. They also used a low salt phosphate buffer and a similar pre-treatment of Aβ42. In contrast to our study, the oligomeric, disc-like particle they described has a size (diameter 10–15 nm, height ~2 nm) that would lead to a sedimentation coefficient of ~6 S, which is much larger than our species of ~2.6 S. Therefore our penta- to hexamer has to be a different oligomeric species. These differences might be caused by a deviating peptide conformation and/or packing. Furthermore we always observe a considerable amount of free monomer in the presence of our oligomeric species, which appears to be absent in the aforementioned study. A study on oligomeric species of Aβ based on high resolution atomic force microscopy also confirms the hexamer and 12 mer as dominant species in addition to monomers and dimers^61^. In earlier studies, small Aβ assemblies with 2 to 6 monomeric units have been identified mostly on the basis of SDS-PAGE analysis of either cross-linked or non-cross linked samples^20, 62^. In addition, a number of computational studies of Aβ oligomerization revealed the existence of several small assemblies, which considerably vary with regard to secondary structure content and overall appearance^60, 63^. In a study of Aβ42 fused to GroES and ubiquitin and performed at high pH in the presence of urea, Aβ42 assembled into SDS-resistant hexamers and tetramers^64^. This study suggests that only the most stable complexes will occur under the chosen reaction conditions.

Our SANS as well as SV results demonstrate that solutions of Aβ42 do not contain dimers, trimers or tetramers at detectable levels. Under the assumption of a rapid equilibrium between these small oligomers, the lower limit of detection would be increased due to broadening of the peak or missing resolution of single species to ~5% according to SV data simulations. A possible explanation for the appearance of these small species in a number of other studies would be that these species represent fragmentation products of larger species. The smallest detectable species is a penta- to hexamer. The fraction of this species is independent of the total Aβ42 concentration and close to 1% of the sedimentation boundary. Aβ42 penta-/hexamers are a recurring motif in literature and have been reported frequently over the last years (reviewed in refs 32 and 65). They appear not only to exist in solution but might also represent the Aβ42 conformation interacting with lipids or lipid bilayers^66, 67^. Nevertheless, we want to emphasize that our study, for the first time, provides evidence for the Aβ42 penta-/hexamer by two solution based methods without the addition of stabilizers. Other groups that showed the presence of similar small oligomers applied the IMS-MS technique^17, 68^, which required the transfer of ionized molecules and molecular complexes into the gas phase, leaving space for additional events to take place. Nevertheless, it remains reasonable to assume, that, because of the concentration dependency of the aggregation, there is a low concentration at which only dimers and trimers but no larger oligomers are in equilibrium with the monomers. Lowering the total Aβ42 concentration in our experiments has been one of the strategies we followed in order to suppress the conversion of monomers into large aggregates and to increase the fraction of the small oligomeric species. However, we could not detect dimers and trimers under our experimental conditions. These species seem to be less populated than the penta-/hexameric oligomer. Additionally, it might be possible to speculate that their higher abundance in other studies is a consequence of fragmentation of larger oligomers.

Our previous studies by sedimentation velocity centrifugation revealed the power of the method to characterize Aβ oligomeric species in equilibrium in solution. The formerly observed gap in the size distribution could be further restricted. We interpreted this gap as indicative for the size, i.e., *s*-value, range of a so called nucleus of aggregation according to the assumption of a nucleated aggregation mechanism. A nucleus would be the smallest assembly, which favours the addition of further monomers or oligomers to facilitate fibril formation versus disassembly. Therefore a nucleus is a kinetically unstable intermediate, which makes it elusive to experimental detection. The hydrodynamic radius of the nucleus has been estimated to be between 5 and 50 nm according to fluorescence correlation spectroscopy measurements performed by Garai and colleagues^69^. This boundary is similar to our estimated size region between 1 and 4 S, although the size of our determined penta-to hexameric species with an *R*_H_ between 2.3 and 2.8 nm is clearly smaller. Nevertheless, our characterized oligomeric species resembles more likely a structure formerly defined as paranucleus as a building block for larger oligomeric species, like the 12- and 18 mers^15, 16^.

In the current study, two independent, solution based methods, which do not necessitate the addition of surfactants, crosslinking or surface interactions, have been combined for the first time to investigate the smallest detectable oligomers occurring during Aβ42 aggregation. It would be desirable to verify, whether these penta-/hexameric species exhibit the primary toxicity agent that leads to synapse failure and finally to memory loss. To address this question larger quantities of this species have to be obtained, which is our goal for future studies.

## Methods

### Experimental details

#### Amyloid β-Proteins

Synthetic Aβ42 was purchased from Bachem as a trifluoroacetate salt (Bachem H-1368). Aβ42 was first dissolved in 100% 1,1,1,3,3,3-hexafluoro-2-propanol (HFIP) for at least 12 h in order to remove any preexisting aggregates. Before usage HFIP was removed by lyophilisation. Aliquots were stored at −80 °C until use. Repeated freeze thaw cycles were generally avoided.

For fluorescence detection measurements we used recombinantly produced Aβ42 with an additional cysteine residue at position 0 (Cys_0_). Cloning was based on a plasmid encoding wild-type Aβ(1–42) as a fusion protein with an N-terminal His_6_-tag, a solubilizing fusion partner (NANP)19 and a modified tobacco etch virus protease (TEV) recognition site, which was kindly provided by Finder, Glockshuber and co-workers^70^. Cys_0_-Aβ42 was labelled by maleimide chemistry with AlexaFluor488 (Molecular Probes, Thermo Fisher Scientific) including a short linker (CH_2_)_5_. The dye conjugate was further purified by SEC from unincorporated dye and is named AF488-Aβ42 (see Fig. S1 for structural information). The partial specific volume of the dye-protein conjugate was calculated according to Durchschlag^71, 72^ as $\bar{v}=0.7127$ cm^3^/g; see also $\bar{v}$ values summarized (Table S1).

A synthetic, covalently linked Aβ40 dimer was obtained by using Aβ40 with an additional Cys residue at the N-terminus, Cys-Aβ40 (Bachem H-7368) under oxidizing conditions. The disulphide bridge linked dimer was purified by high performance liquid chromatography (HPLC). The partial specific volume calculated for the Cys-Aβ40-dimer with the help of sednterp (vs. 20120828 BETA)^73^ is $\bar{v}$ = 0.7316 cm^3^/g at 20 °C and $\bar{v}$ = 0.7295 cm^3^/g at 15 °C.

### Sedimentation Velocity Centrifugation

AUC was performed with an Optima XL-A or ProteomLab XL-A (Beckman Coulter). The ProteomLab XL-A is equipped with standard absorbance optics and an additional fluorescence detection system (Aviv Biomedical Inc.). Samples were filled either in 12 mm titanium double sector cells with 400 µl filling volume (Nanolytics) for absorbance detection or in 3 mm titanium double sector cells with 100 µl filling volume for fluorescence measurements. An integrated spacer places the sample volume to the upper third of a cell assembly guaranteeing an optimal focus position (Nanolytics) for the fluorescence detection. For both cell assemblies quartz windows were used. Labelled or unlabelled Aβ42, pre-treated with HFIP, was dissolved in 10 mM sodium phosphate buffer (NaP*i*), pH 7.4 at concentrations between 0.1 to 160 µM directly prior to the SV experiments. All samples were thermally equilibrated within the centrifuge for ~2 h before starting the run. Sedimentation velocity runs with absorbance detection were performed either at 50,000 rpm or at 60,000 rpm, equivalent to 201,600 *g* or 289,000 *g*, respectively, at the maximum radius of 7.2 cm. At concentrations above 20 µM the runs were performed at 10 or 15 °C to suppress aggregation during centrifugation. For absorbance data collection a radial resolution of 30 µm was chosen together with the shortest possible scan interval, which was ~1.5 min. The detection wavelength was chosen such that sample absorbance was between 0.5 and 1.2 OD. In general, up to 25% loss of Aβ42 concentrations had been determined by comparing the signal of the first scan with the spectroscopically determined concentrations of the samples prior to filling the samples into the cells for SV measurements. This loss during the acceleration phase might be either due to incomplete dissolution, rapid aggregate formation, or attachment of Aβ42 to surfaces, i.e. glass windows. Higher recovery rates were not achievable with Aβ at neutral pH. It is a special feature of AUC that these losses can be easily quantified.

For the fluorescence detection system, based on laser excitation at 488 nm, the radial resolution was fixed at 20 µm and data acquisition was not done sequentially but simultaneously for all sample sectors, leading to scan intervals shorter than 1 min. Each sample sector was measured with an individually adjusted signal amplification factor resulting in 2000−3400 relative fluorescence units (RFU). The addition of carrier protein BSA, as recommended for fluorescence detection, was not appropriate for our system because Aβ directly interacts with BSA^74^. Low percentages of Tween 20 did not alter the signals and were therefore not applied either. All SV data was initially analysed using a continuous distribution c(s) with maximum entropy. Thereafter the *c*(*s*)-distribution analysis with the size-distribution option “with prior probabilities” was applied as implemented in the software package sedfit (vs. 14.7 g; May 2015) (http://www.analyticalultracentrifugation.com/)^75^. For data analysis a resolution of 0.05 S with a confidence level (F-ratio) of 0.95 was chosen for the appropriate *s*-value range. A more detailed description of the fitting process has been added to the supplement. All presented *c*(s) distributions have root mean square deviations of <1% of the total signal. Sedimentation coefficients are reported as *s*_20,w_ values in Svedberg units with 1 S = 10^−13^ s, which represent the apparent sedimentation coefficient normalized to the standard conditions of 20 °C and pure water solvent.

#### Small Angle Neutron Scattering

Proteins were dissolved in HFIP for three weeks and dried before being resolved in deuterated buffer (50 mM Nap*i*, pH 7.4 + 150 mM NaCl in 100% D_2_O, filtered with Anopore Whatman, pore size 20 nm). Samples were incubated at 7 °C at rest. SANS experiments were performed at the small angle diffractometer, KWS-2 of Heinz Maier-Leibnitz Zentrum (MLZ Garching, Germany) with wavelengths between 0.47 nm and 0.81 nm and detector distances from 1.1 m to 19.7 m to cover a wave vector range of 0.001 Å^−1^ to 0.5 Å^−1^ ^76^. The wavelength spread was ${\rm{\Delta }}\lambda /\lambda =0.2$ and measurement times from 1 to 4 h dependent on detector distance and sample scattering were used. Samples and buffer measurements for background correction were performed in quartz cells of 2 mm thickness together with appropriate measurements for detector sensitivity and dark current. Appropriate standard methods for evaluation and background correction were used from the software QtiKWS^77^.

### Computational details

#### Molecular Dynamics calculations

Five independent all-atom MD simulations of twenty Aβ42 monomers inserted in a cubic box with a side length of 350 Å and periodic boundary conditions were performed. We applied different initial velocity distributions for each simulation and simulated in total 2.5 μs, with 500 ns per simulation. We used the parallel processing MD software Gromacs 4.5.5 for performing the simulations with a leap-frog stochastic dynamics integrator, the OPLS/AA force field^78–80^, and implicit solvent using a Generalized Born model with a hydrophobic solvent accessible surface area term (GBSA)^81^. The temperature coupling was done via velocity rescaling with a stochastic term algorithm^82^ using a time constant for coupling of 2 ps and keeping the system at 300 K. Electrostatic interactions were treated with the cut-off method with a value of 1.2 nm and van der Waals interactions were also cut at 1.2 nm. Hydrogen atoms were treated as virtual interaction sites, permitting an integration time step of 4 fs while maintaining energy conservation^83^. From the 5 × 500 ns simulations we extracted all tetramer, pentamer and hexamer conformations and clustered all structures for each oligomer size using the method of Daura and colleagues^84^ with a cut-off of 0.2 nm. For the resulting cluster centres we calculated sedimentation coefficients using the program HydroPro^85^. To this end, we first estimated the partial specific volume as the total protein volume divided by the molecular weight of the protein. To estimate the total protein volume we used the program 3 V Volume Calculator^86^, and for determining the molecular weight of the protein we used VMD^87^.

## Electronic supplementary material

Supplementary info
