# Supplementary material for: Aβ42 pentamers/hexamers are the smallest detectable oligomers in solution
Source: Sci Rep. 2017 May 30;7:2493. doi: 10.1038/s41598-017-02370-3 (PMC5449387; doi:10.1038/s41598-017-02370-3)
Supplement: Supplementary file 1 — Supplementary info [file 41598_2017_2370_MOESM1_ESM.pdf]

Aβ42 pentamers/hexamers are the smallest detectable oligomers in solution.

Martin Wolff[1,2,†], *Bo Zhang-Haagen*[2,3], *Christina Decker*[1], *Bogdan Barz*[2], *Mario Schneider*[1], *Ralf Biehl*[3,4], *Aurel Radulescu*[4], *Birgit Strodel*[2], *Dieter Willbold*[1,2], *and Luitgard Nagel-Steger*[1,2,*]

[1] Institut für Physikalische Biologie, Heinrich-Heine-Universität Düsseldorf, 40225 Düsseldorf, Germany

[2] Institute of Complex Systems, Structural Biochemistry (ICS-6), Forschungszentrum Jülich, 52425 Jülich, Germany

[3] Jülich Centre for Neutron Science & Institute of Complex Systems, Neutron Scattering (JCNS-1&ICS-1), Forschungszentrum Jülich, 52425 Jülich, Germany,

[4] Jülich Centre for Neutron Science, Outstation at MLZ (JCNS-MLZ), Forschungszentrum Jülich, 85747 Garching, Germany

SUPPORTING INFORMATION

Partial specific volumes of Aβ peptides

Table S1 lists the partial specific volumes of the amyloid beta peptides used in this study. All values for peptides had been calculated by using the software SEDNTERP[1] and for dye conjugated peptides by additionally summing up partial specific volumes of molecular fragments according to Durchschlag & Zipper[2].

Table S1: Partial specific volumes and molar masses of the Aβ-peptides used in this study.

	Molar mass (g/mol)	$\bar{v}$ at 20°C (cm^3/g)
Aβ42	4514.10	0.7377
AF488-Aβ42	5337.77	0.7127
FITC-β-Ala-Aβ42	4974.57	0.7350
Aβ40	4329.87	0.7341
Cys-Aβ40	4433.01	0.7316
Cys-Aβ40-dimer	8864.00	0.7288

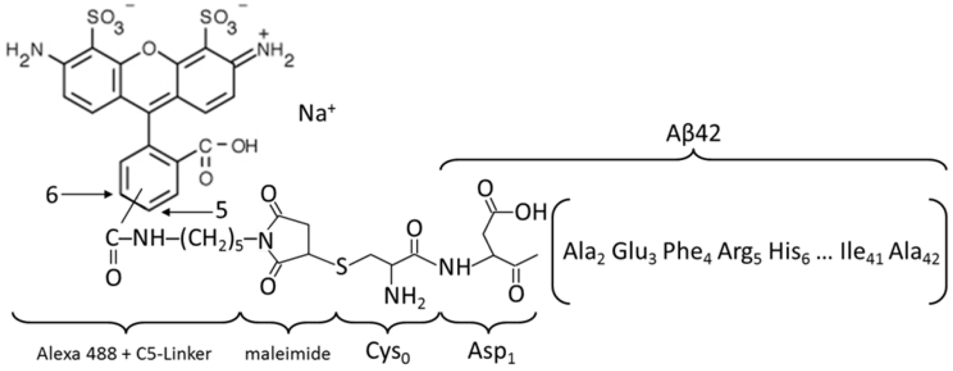

Figure S1: Structure of the N-terminally Alexa 488 labeled Aβ42. The Alexa fluorophore and the N-terminal aspartate of Aβ42 are separated by a C5 linker, the maleimide group, and the cysteine residue. The bond pointing into the phenyl group between C5 and C6 indicates the possibility of two isomeric forms.

Detectability of a dimer by SV analysis

The software package UltraScan II (vs 9.9; rev. 1927) (http:\\www.ultrascan.uthscsa.edu) offers a simulation tool based on finite element (ASTFEM) methods for the calculation of radial sedimentation profiles as a function of time for single or multiple components [3]. Beforehand, the components have to be modelled with regard to their mass, shape and partial specific volume. Afterwards, the calculated sedimentation profiles can be processed by data evaluation software like SEDFIT in order to retrieve for example a $c(s)$ distribution. A model for two ideal, non-interacting components was chosen for monomeric and dimeric cys-Aβ40. To evaluate whether in SV analysis it is possible to specifically detect the dimer we simulated a particle with the double mass of Aβ42 and a slightly more compact shape than the monomer with a theoretical *s*-value between 1.0 and 1.5 S. Assuming a composition of 50 % monomer and 50 % dimer as independent species at 20°C in sodium phosphate buffer the dimer is clearly distinguishable from the monomer by sedimentation velocity centrifugation at maximum speed (289,000g). Experimentally this could be verified by analysing a covalently linked Aβ40 dimer, which was generated by introducing a disulphide bridge between a cysteine engineered at the N-terminus. Data evaluation reveals a difference in *s*-value of 0.25 S between the monomer and the dimer (Fig. S2).

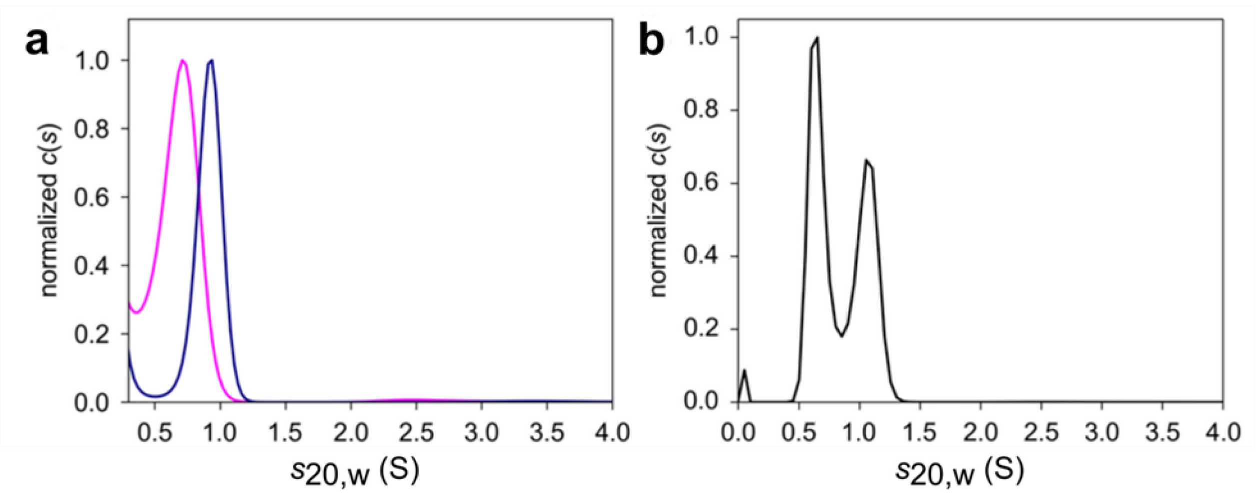

Figure S2: SV analysis of Aβ40 monomer and dimer at 60,000 rpm, 15°C. (**a**) Comparison of monomeric Aβ40 ($s_{20,w}$ = 0.65 S), (magenta) and dimeric Aβ40 (dark blue), which had been covalently linked by a disulphide bridge ($s_{20,w}$=0.9 S). (**b**) c(s) distribution obtained from simulated SV data for a 1:1 mixture of monomeric Aβ40 and dimeric Aβ40, decorated with 0.5 % random noise, 0.02 % radial invariant, and 0.02 % time invariant noise. The frictional ratio was assumed 1.5 for the monomer and 1.4 for the dimer. The *c*(*s*) distributions were normalized according to maximum peak height.

Detailed description of SV data fitting procedure

The fitting of SV data was performed with SEDFIT (14.7g; May 2015) according to the procedure reported in Wafer et al. 2016.[4] The following steps were worked through for the SV data derived from fluorescence detection of 100 nM AF488-Aβ42.

1) Generation of a normal *c*(*s*)

   At least 100 scans, which covered the whole sedimentation process, were used. Parameters were the same for all data sets, with s_{min}=0 and s_{max}=5 S, resolution 251, resulting in 0.02 S effective resolution. The meniscus and bottom positions, baseline, time invariant noise and frictional ratio were allowed to float.

2) Manual Bayesian analysis, $c^{MP}(s)$

This option is found in sedfit under size-distribution options-use prior probabilities. The values for s-value and amplitude, which were entered into the table, where those obtained from initial $c(s)$. The peak width was set to 0.1 S. Whenever these starting values had been changed, the data was first re-fit to generate the normal $c(s)$.

3) Non-interacting discrete species model and F-statistics

This model allows the entries for four independent species. For our data only three species were required. Concentration of the first species (most prominent species) initialized to total optical signal and for the other species with values relative to this first species, e.g. 5 % results in 0.005. The s-value and molar mass was again taken from c(s); meniscus position and time invariant noise were floated.

Then the critical value of root mean square deviation (rmsd) was determined using the F-statistics calculator in sedfit: Options – statistics - calculate variance ratio (F-statistics).

The default values for confidence level; first, second degrees of freedom were used.

In order to avoid trapping of the fit process in local minima Marquardt-Levenberg (ML) and simplex global minimization algorithm were used alternately on each data set until no further change in rmsd was observable. At this point three further MLs were added to test for stability of the solution. Individual species were defined to be statistically significant if their removal from the fit (deletion from the table of non-interacting species) resulted in an rmsd larger than the before determined critical rmsd.

Atomic force microscopy of FITC-β-Ala-Aβ42

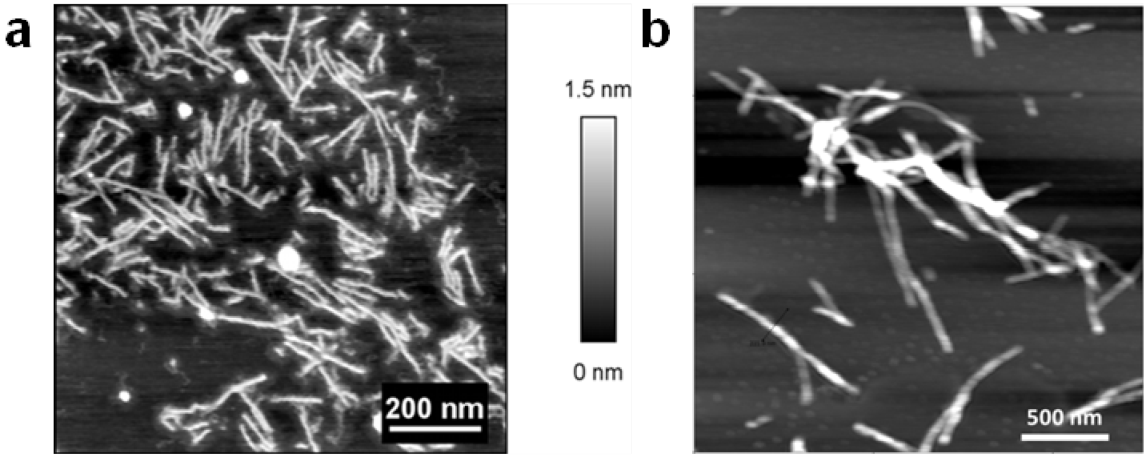

Figure S3: (**a)** Detection of FITC-β-Ala-Aβ42 fibrils by atomic force spectroscopy. FITC-β-Ala-Aβ42 (1 µM) had been incubated for 5 days at 37 °C. Afterwards the sample had been imaged by atomic force microscopy in air on mica surface. The greyscale on the right side indicates height from black to white. (**b)** For comparison, Aβ42 fibrils (80 µM) after 4 days preincubation in 10 mM NaPi, pH7.4 at RT and 600 rpm. Diluted samples had been air dried on mica before analysis by tapping mode in air.

In order to demonstrate that the fluorophore labelled Aβ peptide was capable of fibril formation a solution of 1 µM FITC-β-Ala-Aβ42 had been incubated in phosphate buffered saline, pH 7.4 for 5 days at 37°C. Forty-microliter aliquots of the samples were transferred to freshly cleaved mica. After 30 min to 40 min incubation the mica surface was washed three times with 100 µl deionized water (Millipore). Excess water was removed with compressed N_2. Imaging of FITC-Aβ42 was performed with Nanowizard II (JPK Instruments AG) in intermittent contact in air[5], using standard silicon cantilevers (OLYMPUS OMCL-AC160TS). In AFM fibrils with rather uniform diameter and 100 to 200 nm length were detected (Fig. S3a). For comparison amyloid fibrils formed in a sample of unlabelled Aβ42 are shown in Fig. S3b.

Dynamic light scattering in parallel to SANS measurements

Samples used for SANS were in parallel subjected to dynamic light scattering measurements. Experiments were performed with a Zetasizer Nano ZS (Malvern Instruments, Worcestershire, UK). The instrument uses a He-Ne laser with $\lambda = 632.8$ nm and vertical polarization in backscattering geometry at 173°. The sample cell is a UV-Cuvette micro (BRAND, Wertheim, Germany) with 70 µl sample volume. To cover also larger aggregates the measurements duration was set to 1h to get reliable data at long times. The auto correlation function was analyzed by non-negative least square (NNLS) algorithm[6] followed by protein analysis (L-curve)[7] as implemented in the instrument software. Figure S4a shows the correlation functions with the resulting distribution of relaxation times after 0.5 h incubation at 7 °C for the three different Aβ42 concentrations also studied by SANS. Surprisingly, we observe for the lowest Aβ42 concentration the slowest relaxation indicating the largest aggregates. This effect might be caused by a faster aggregation at higher Aβ42 concentrations leading to aggregates that are large enough to sediment before the DLS measurement. The relaxation time distributions from L-curve analysis show three populations. For the higher concentrations we observe 3 populations analogously in size: low molecular weight oligomers (LMW) with $R_H \approx 4$ nm, middle molecular weight oligomers (MMW) with $R_H \approx 11$ nm and high molecular weight oligomers (HMW) with $R_H \approx 70$ nm. For the lowest concentration we observe two populations of much larger size ($R_H > 100$ nm) and one population around the average size of the populations observed at higher concentration. In case of the lowest concentration the correlation function is dominated by the contribution of the larger aggregates. The L-Curve analysis, in the same way as the well-known CONTIN analysis[8], tries to minimize the number of populations resulting in only one population describing the smaller aggregates as one population of minor contribution to the overall signal.

We observe the remaining populations, which contribute stronger to the signal allowing the L-curve analysis to resolve details of the contributing populations.

Monomers as observed in SEC measurements have a hydrodynamic radius of about 1 nm[9]. Monomers cannot be separated from the contribution of LMW because of the small scattering contribution even if their concentration might be large as the scattered intensity is proportional to R^6. On the other side for a separation of species in DLS hydrodynamic radii need to be separated by a factor of 5, which is not given for monomers and LMW oligomers. The HMW oligomers contribute a factor 4 more to the scattered intensity than the LMW oligomers despite their extreme low concentration (<1% volume fraction) because of their huge size. Increasing the concentration to 1 mg/ml the MMW species grows to 33% (intensity fraction), which was also observed in prior research3. With more than 80% v/v the LMW oligomers are the main component of the oligomers (Fig. S4b).

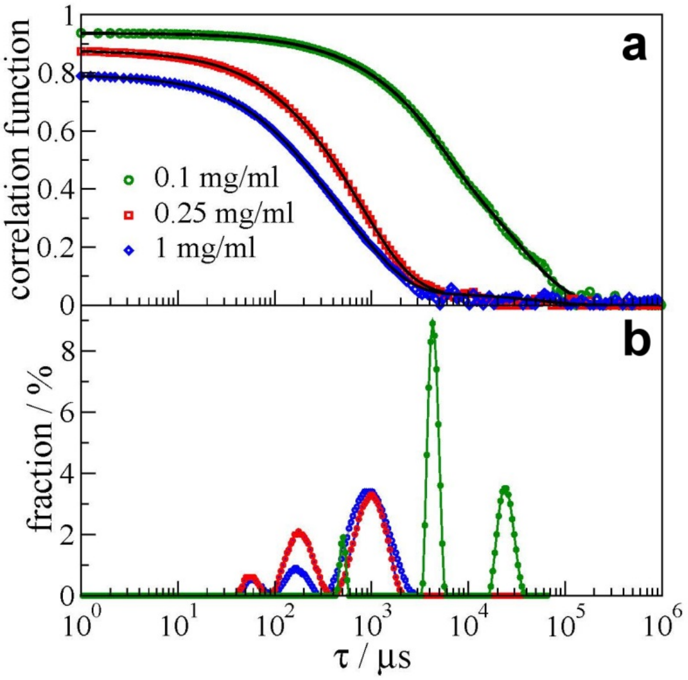

Figure S4: DLS experiments of Aβ1-42 after 0.5 h incubation at 7°C. (**a**) Correlation functions with the result of the L-Curve analysis as black line over the data. (**b**) Intensity weighted relaxation time distribution with colours as in a). X-scales of a) and b) are the same.

Calculation of hydrodynamic radii for different oligomers based on determined sedimentation coefficients

For comparability, hydrodynamic radii for three different oligomers, the tetramer, pentamer and hexamer have been calculated assuming a hydration of 0.398 g H_2O/g protein. Equation (S1) is obtained from the Svedberg equation by insertion of the expression for the frictional coefficient $f = 6\pi\eta R_H$ according to Stokes' law.

$$R_H = m(1 - \bar{v}\rho)/6\pi\eta s \tag{S1}$$

with $m = \left(nM_p + \delta n M_p\right)/N_A$ and $\bar{v} = \left(M_p\bar{v}_p + \delta M_p \bar{v}_h\right)/\left(M_p + \delta M_p\right)$

R_H	hydrodynamic radius
m	mass of hydrated oligomer build from *n* monomers
ρ	water density at 20°C: 0.99823g/cm^3
η	water viscosity at 20°C: 0.01002 Poise
s	sedimentation coefficient in water at 20 °C
M_P	molar mass of protein
δ	estimated fraction of bound hydration water, here δ = 0.398 g H_2O/g protein [10-12]
N_A	Avogadro constant
$\bar{v}_p$	partial specific volume of protein (index p)
$\bar{v}_h$	partial specific volume of hydration shell (index h)

The density of hydration shell water is assumed to be 10 % higher than bulk water density[13].

Table S2: Calculated hydrodynamic radii for the different oligomeric states based on experimentally determined sedimentation coefficient.

Given:	**Aβ42**	**MW (g/mol)**	**R_H (nm)**
**$s_{20,w}$ = 2.56 S**	tetramer	1.8056E+04	1.85
	pentamer	2.2571E+04	2.31
	hexamer	2.7085E+04	2.77
	**AF488-Aβ42**	**MW (g/mol)**	**R_H (nm)**
**$s_{20,w}$ = 2.32 S**	tetramer	2.1351E+04	2.63
	pentamer	2.6689E+04	3.28
	hexamer	3.2027E+04	3.94

Determination of molar mass *M* based on scaling law:

N_A is the Avogadro constant $6.022 \cdot 10^{23}$ mol^{-1}, ρ the density of water at 20 °C (0.998 g/cm³), η the viscosity of water at 20 °C (0.010019 Poise), $\bar{v}$ the partial specific volume (0.7377 cm³/g) of Aβ42.

$$\left(\frac{M}{N_A}\right)^{2/3} = \frac{s \cdot f/f_0}{1-\bar{v}\rho} 6\pi\eta \left(\frac{3\bar{v}}{4\pi}\right)^{1/3}$$

For a particle with sedimentation coefficient $s_{20,w}$=2.56 S and frictional ratio f/f_0=1.2 the molar mass calculated with the above equation is 26,100 g/mol. This is in agreement with the pentamer/hexamer of Aβ42.

Sedimentation velocity analysis in the presence of 150 mM sodium chloride

In order to investigate that the different ionic strength conditions lead to comparable Aβ42 size distributions in SV analysis we analysed 1 µM FITC-β-Ala-Aβ42 in phosphate buffer with 150 mM NaCl (Fig. S5). The truncated peak at 0.3 S is caused by free fluorophore. The oligomer fraction is slightly higher than at lower salt conditions. Nevertheless the oligomer

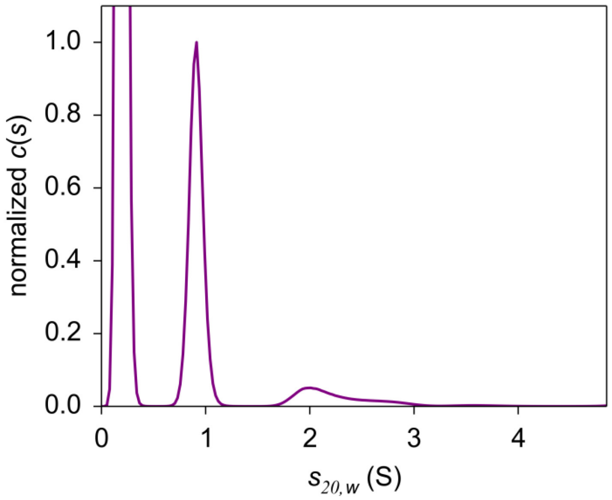

Figure S5: SV analysis of 1 μM FITC-β-Aβ42 in phosphate buffer with 150 mM NaCl, pH 7.4; at 20°°C and 60,000 rpm. Normalization with regard to peak height of monomeric FITC-β-Aβ42.

REFERENCES

1 Hurton, T. *et al. SEDNTERP*, <http://bitcwiki.sr.unh.edu/index.php/Main_Page> (2016) (Date of access: 04/01/2017).

2 Durchschlag, H. & Zipper, P. in *Progress in Colloid & Polymer Science: Ultracentrifugation* Vol. 94 (ed G. Lagaly M.D. Lechner (guest editor) F. Kremer) Ch. Calculation of the partial volume of organic compounds and polymers, 20-39 (Springer Verlag Berlin Heidelberg GmbH, 1994).

3 Cao, W. & Demeler, B. Modeling analytical ultracentrifugation experiments with an adaptive space-time finite element solution of the Lamm equation. *Biophys J* **89**, 1589-1602, doi:10.1529/biophysj.105.061135 (2005).

4 Wafer, L., Kloczewiak, M. & Luo, Y. Quantifying Trace Amounts of Aggregates in Biopharmaceuticals Using Analytical Ultracentrifugation Sedimentation Velocity: Bayesian Analyses and F Statistics. *Aaps J* **18**, 849-860, doi:10.1208/s12248-016-9925-y (2016).

5 Serem, W. K., Bett, C. K., Ngunjiri, J. N. & Garno, J. C. Studies of the growth, evolution, and self-aggregation of beta-amyloid fibrils using tapping-mode atomic force microscopy. *Microsc Res Tech* **74**, 699-708, doi:10.1002/jemt.20940 (2011).

6 Morrison, I. D., Grabowski, E. F. & Herb, C. A. Improved techniques for particle size determination by quasi-elastic light scattering. *Langmuir* **1**, 496-501, doi:10.1021/la00064a016 (1985).

7 Calvetti, D., Reichel, L. & Shuibi, A. L-Curve and Curvature Bounds for Tikhonov Regularization. *Numerical Algorithms* **35**, 301-314, doi:10.1023/b:numa.0000021764.16526.47 (2004).

8 Provencher, S. W. CONTIN: A general purpose constrained regularization program for inverting noisy linear algebraic and integral equations. *Computer Physics Communications* **27**, 229-242, doi:http://dx.doi.org/10.1016/0010-4655(82)90174-6 (1982).

9 Zhang-Haagen, B. *et al.* Monomeric amyloid beta peptide in hexafluoroisopropanol detected by small angle neutron scattering. *PLoS One* **11**, e0150267, doi:10.1371/journal.pone.0150267 (2016).

10 Kuntz, I. D. Hydration of macromolecules. IV. Polypeptide conformation in frozen solutions. *J Am Chem Soc* **93**, 516-518 (1971).

11 Pessen, H. & Kumosinski, T. F. Measurements of protein hydration by various techniques. *Methods Enzymol* **117**, 219-255 (1985).

12 Kuntz, I. D., Jr. & Kauzmann, W. Hydration of proteins and polypeptides. *Adv Protein Chem* **28**, 239-345 (1974).

13 Svergun, D. I. *et al.* Protein hydration in solution: experimental observation by x-ray and neutron scattering. *Proc Natl Acad Sci U S A* **95**, 2267-2272 (1998).
